# Distinguishing between the metabolome and xenobiotic exposome in environmental field samples analysed by direct-infusion mass spectrometry based metabolomics and lipidomics

**DOI:** 10.1007/s11306-014-0693-3

**Published:** 2014-07-15

**Authors:** Andrew D. Southam, Anke Lange, Raghad Al-Salhi, Elizabeth M. Hill, Charles R. Tyler, Mark R. Viant

**Affiliations:** 1 School of Biosciences, University of Birmingham, Edgbaston, Birmingham, B15 2TT UK; 2 Biosciences, College of Life and Environmental Sciences, University of Exeter, Exeter, EX4 4QD UK; 3 School of Life Sciences, University of Sussex, Falmer, Brighton, BN1 9QG UK

**Keywords:** *Rutilus rutilus*, Exposome, Xenometabolome, Endometabolome, Lipidome

## Abstract

Environmental metabolomics is increasingly used to investigate organismal responses to complex chemical mixtures, including waste water effluent (WWE). In parallel, increasingly sensitive analytical methods are being used in metabolomics studies, particularly mass spectrometry. This introduces a considerable, yet overlooked, challenge that high analytical sensitivity will not only improve the detection of endogenous metabolites in biological specimens but also exogenous chemicals. If these often unknown xenobiotic features are not removed from the “biological” dataset, they will bias the interpretation and could lead to incorrect conclusions about the biotic response. Here we illustrate and validate a novel workflow classifying the origin of peaks detected in biological samples as: endogenous, xenobiotics, or metabolised xenobiotics. The workflow is demonstrated using direct infusion mass spectrometry-based metabolomic analysis of testes from roach exposed to different concentrations of a complex WWE. We show that xenobiotics and their metabolic products can be detected in roach testes (including triclosan, chloroxylenol and chlorophene), and that these compounds have a disproportionately high level of statistical significance within the total (bio)chemical changes induced by the WWE. Overall we have demonstrated that this workflow extracts more information from an environmental metabolomics study of complex mixture exposures than was possible previously.

**Electronic supplementary material:**

The online version of this article (doi:10.1007/s11306-014-0693-3) contains supplementary material, which is available to authorized users.

## Introduction

The popularity of metabolomics for analysing biological specimens sampled directly from the field is rapidly increasing, with the metabolic phenotype of the organism serving as an indicator of the responses to stress caused by climate change, ocean acidification and pollution (Van Aggelen et al. 2010; Hines et al. 2010; Southam et al. 2011; Flores-Valverde et al. 2010; Bundy et al. 2009). This popularity is anticipated to escalate as metabolomics becomes a more routine tool for environmental monitoring. Across the discipline of metabolomics, whether applied to the environmental, biological or biomedical sciences, there is a constant drive towards increasing the analytical sensitivity of the technology to detect greater numbers of endogenous metabolites in an organism. This is evidenced by the rising use of mass spectrometry (MS) as the leading technology for metabolomics (Zhang et al. 2012), which includes the established high-resolution direct infusion (DI) MS approach (Draper et al. 2013; Han et al. 2008; Lin et al. 2010; Southam et al. 2007, 2011; Zhang et al. 2014). However, in the field of environmental metabolomics in particular, this introduces a considerable and yet overlooked challenge. The higher analytical sensitivity will not only increase our ability to detect endogenous metabolites, but also xenobiotic compounds. For laboratory-based environmental metabolomics studies in which organisms are typically exposed to defined xenobiotic chemicals, it is relatively straightforward to remove any signals arising from these compounds prior to analysis of the biological datasets. However, for field-based metabolomics studies, the organisms that are studied are typically exposed to complex mixtures of unknown xenobiotics, which leads to detection of both the endogenous organism metabolome (termed endometabolome) and what has been referred to as the exposome or xenometabolome (Al-Salhi et al. 2012; Holmes et al. 2007). In fact the xenometabolome should be further sub-categorised into the unmodified exogenous chemicals (which here we term ‘unmodified xenobiotics’) and those exogenous compounds that are metabolised within the organism (‘metabolised xenobiotics’). It is therefore important to identify whether the detected features are of endogenous or xenobiotic origin in order to correctly interpret data from a metabolomics analysis of field-derived specimens. This approach will enable the significant endogenous metabolic responses to a complex xenobiotic mixture to be interpreted correctly, while also allowing important information on the accumulation and metabolism of xenobiotics in the organism under investigation.

In this study we describe a novel computational ‘module’ for the DIMS SIM-stitch processing pipeline (Southam et al. 2007) that can distinguish between endogenous and xenobiotic peaks in metabolomics datasets. Our approach has been developed and applied to testes sampled from a male freshwater fish (*Rutilus rutilus*) exposed to different concentrations of waste water effluent (WWE) outflow from a sewage treatment works. WWE is highly complex, containing tens of thousands of chemicals, and is known to impact adversely on fish reproductive health in rivers, often effected via alterations to the endocrine system (Al-Salhi et al. 2012). Our approach requires that DIMS measurements are made on both the biological specimens (extracts of roach testes tissue in this study) and the complex WWE. The (typically unidentified) peaks in the mass spectra arising from the exogenous chemicals in the WWE were modified to take into account selected common metabolic reactions that the xenobiotics can undergo (e.g. phase two conjugation), and then compared to the mass spectra of the roach testes extracts. This approach enables peaks to be classified as endogenous, unmodified xenobiotics, or metabolised xenobiotics, and therefore the analysis of the effects of the WWE on the actual endogenous biochemistry of the fish. We subsequently validated this novel workflow by confirming the identities of selected xenobiotic peaks using ultra-high pressure liquid chromatography time-of-flight mass spectrometry (UHPLC-QTOF MS) (Flores-Valverde and Hill 2008; Al-Salhi et al. 2012).

## Methods

### Fish maintenance and WWE exposure

The wastewater treatment works chosen for this study received influent from a population equivalent of 142,370 and >99 % of the influent was from domestic sources. In the treatment works the influent was treated by fine bubble diffusion activated sludge and trickling filters processes. The effluent has been shown previously to contain a spectrum of endocrine disrupting chemicals and to induce feminising effects in exposed fish (Al-Salhi et al. 2012; Liney et al. 2006). A mixed-sex population of roach were used (Calverton Fish Farm, UK Environment Agency) that were 2 + years in age (approximately 1 month before spawning and derived from wild parental stock) with mean ± SEM length of 10.9 ± 0.2 cm and weight 23.0 ± 1.2 g. Fish were exposed in duplicate tanks (20 fish per tank) to 100 % WWE, 50 % WWE diluted with charcoal- and UV-treated tap water or charcoal- and UV-treated tap water (subsequently referred to as dilution water). The fish were exposed for 28 days in 1 m^3^ tanks under continuous flow-through conditions, ambient temperature and photoperiod and were fed frozen gamma-irradiated brine shrimp (*Artemia* sp.) or bloodworm (*Chironomus* sp.). Feeding was withheld 24 h prior to sampling. The flow rates through each tank were 5 L/min and all tanks were aerated to ensure sufficient oxygen supply. At the end of the exposure period, the fish were terminally anesthetized, the gonads were dissected rapidly and a subsample of each gonad was fixed for histological analysis to confirm sex as reported earlier (Southam et al. 2011) and the remainder of the gonad was frozen on dry ice and stored at −80 °C until analysis. Only testes were used for further analyses.

### Metabolite, lipid and steroid extraction from tissues

The roach testes samples (200–500 mg) were homogenised in 8 µL/mg methanol using a bead based homogeniser (Precellys-24, Stretton Scientific) and split for further sample preparation for non-targeted metabolomics (80 µL aliquot) and steroid analysis (remainder of homogenate). Prior to steroid extraction 2 ng of deuterated d3-testosterone and 4 ng of deuterated d4-cortisol were added to the methanol homogenate and then the steroids were extracted by two sequential solid phase extractions (Flores-Valverde and Hill 2008; Southam et al. 2011). For the non-targeted direct infusion mass spectrometry (DIMS) metabolomics and lipidomics, the metabolites/lipids were extracted from the aliquot using a two-step methanol:chloroform:water method with a final solvent ratio of 2:2:1.8, respectively (Wu et al. 2008). Extract blanks were produced by the same protocol but in the absence of tissue. 200 µL of the extracted polar phase and 100 µL of the non-polar phase were dried and resuspended in either 80:20 methanol:water containing 20 mM ammonium acetate (polar metabolites) or 2:1 methanol:chloroform containing 5 mM ammonium acetate (lipids).

### WWE sample preparation

Water samples (500 mL) were taken from each treatment and stabilised with methanol (3 % final concentration) and acetic acid (1 % final concentration). The dilute nature of the samples required them to be concentrated using pre-conditioned solid-phase cartridges (Sep-Pak C18, Waters Ltd.). The cartridges were dried under a stream of nitrogen and stored at −20 °C until analysis. Columns were washed twice with 1 mL HPLC grade water and then analytes were eluted with 1 mL HPLC grade methanol. Sample was dried under nitrogen and re-suspended in 80:20 methanol:water containing 20 mM ammonium acetate. This preparation method is different to that used to extract the tissue samples as each method was optimal for its specific sample type. Potential matrix-related variations between sample types have been accounted for in the “Procedure to distinguish endogenous and xenobiotic peaks *”* (Methods) and the “Novel workflow *”* (Results) sections.

### *UHPLC*-*QTOF MS analysis of roach testes extracts and steroid standards*

For quantification of steroids, calibration standards [cortisone, cortisol, androstenedione, 11-ketotestosterone (11-KT) and 11-hydroxyandrostenedione; Steraloids] were prepared and mixed with a pooled testes extract (equivalent of 100 mg testes per sample) and 2 ng d3-testosterone and 4 ng d4-cortisol (internal standards) per UHPLC-QTOF MS injection. Aliquots of pooled testes extract were prepared spiked with internal standards to analyse levels of steroids in the calibration mixture. Roach samples and steroid standards (in 20 µL 50:50 methanol:water) were analyzed by UHPLC-QTOF MS (UPLC-QTOF, Waters, UK), according to our earlier protocols (Flores-Valverde and Hill 2008). The mobile phases were (A) 95 % water, 5 % acetonitrile (ACN), 0.25 % formic acid; and (B) 100 % ACN. The gradient used was 0–14.9 min, from 20 to 80 % B; 14.9–15.0 min, from 80 to 100 % B; 15.0–25.0 min, 100 % B (Southam et al. 2011). The five steroids were quantified by integrating the molecular ion intensity in the chromatograms and comparing to standard curves (MassLynx, Waters).

Identification of chemical contaminants in testes extracts were performed from elemental composition and isotopic fit and, after QTOFMS and collision induced dissociation, comparison of fragmentation patterns with authentic standards or the METLIN database.

### DIMS analysis, data processing and peak annotation

Roach testes extracts (polar and non-polar) were analyzed by DIMS in negative ion mode using the selected ion monitoring (SIM)-stitching method (Southam et al. 2007) on a hybrid linear ion trap/Fourier transform ion cyclotron resonance mass spectrometer (LTQ FT, Thermo Fisher Scientific, Germany). Data was processed and mass calibrated [using calibrants in Table S1—non polar, and reference Southam et al. (2011)—polar] as described previously (Southam et al. 2007). Each sample was analyzed in triplicate and filtered into a single peak list (Payne et al. 2009). The WWE samples were mass calibrated by aligning common background peaks to those from the calibrated testes sample (Table S2). Final matrices containing peak intensity data were each normalized by the probabilistic quotient (PQN) method (Dieterle et al. 2006) prior to statistical analysis. Peaks were annotated from the Kyoto Encyclopedia of Genes and Genomes (KEGG) database using MI-Pack software (Weber and Viant 2010).

### Statistical analyses of DIMS metabolomics and steroid measurements

Principal components analysis (PCA) was conducted using PLS_Toolbox (Eigenvector Research) in MatLab (The MathWorks). Prior to PCA, a generalized log transformation (Parsons et al. 2007) was applied to the PQN normalized DIMS datasets and data was mean centred. Within the normalised DIMS datasets (Polar data: control *n* = 12; 50 % WWE *n* = 15; 10 % WWE *n* = 15. Non-polar data: control *n* = 12; 50 % WWE *n* = 19; 100 % WWE *n* = 20), ANOVA was conducted on each peak across control, 50 % WWE and 100 % WWE treatment groups. A false discovery rate was applied to the *p* values (Benjamini and Hochberg 1995), which were corrected to *q* values (the threshold for significance was *q* < 0.1). Metabolite fold-changes were calculated as the average intensity in a treated group relative to the average of the control group. In the steroid data (control *n* = 15; 50 % WWE *n* = 15; 100 % WWE *n* = 16), ANOVA (one-way with Tukey’s post hoc testing) was conducted to assess changes in steroid levels between the treated and control groups using Minitab 17 (Minitab Inc.).

### Procedure to distinguish endogenous and xenobiotic peaks

**Fig. 1:**
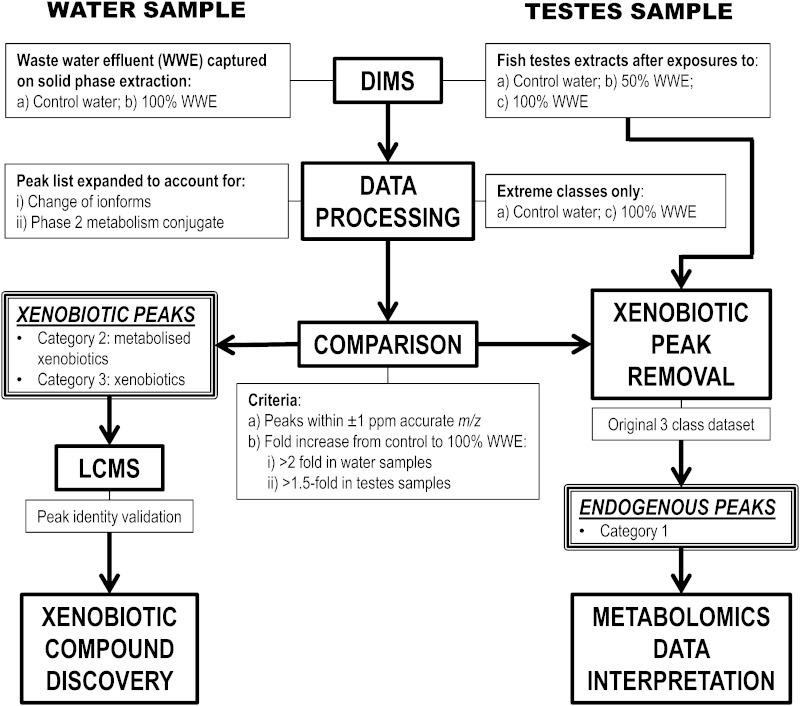
Schematic of novel workflow for comparing DIMS analyses of roach testes extracts and WWE samples to categorise peaks as: (*1*) endogenous metabolome, (*2*) metabolised xenobiotics; and (*3*) unmodified xenobiotics. Prior to comparison with the testes peak (*m/z*) list, the WWE peak list was modified several times to take into account several ion-form changes or metabolic reactions that could have occurred. The workflow was validated by confirming the identities of a selection of peaks predicted as xenobiotic or metabolised xenobiotic using liquid chromatography (LC) MS. Roach had been treated with 100 % WWE, 50 % WWE or dilution (control) water for 28 days

This procedure is discussed and justified more extensively in the “Results and discussion” section and is summarised in Fig. 1. The *m/z* and empirical formulae of the selected common metabolic modifications are as follows: (i) addition of glutathione: +C_10_H_15_N_3_O_6_S, 305.06816 *m/z*; (ii) glucuronidation: +C_6_H_10_O_7_–H_2_O, 176.03209 *m/z*; (iii) sulfation: +SO_3_, 79.95682 *m/z*; (iv) hydroxylation: +OH–H, 15.99491 *m/z*; and (v) addition of methoxy: +OCH_3_–H, 30.01056 *m/z*. To account for ionisation changes between the two different sample types, the following ion-form modifications were used (a) conversion of [M–H] to [M+OAc]: +C_2_H_3_O_2_, 60.02113 *m/z*; (b) conversion of [M–H] to [M+Cl]: +HCl, 35.97668 *m/z*; and (c) conversion of [M–H] to [M+Na–2H]: +Na–H, 21.98194 *m/z*. Calibrated testes extract and water peak lists were compared using a custom written MatLab script (available upon request).

## Results and discussion

### The WWE induces endocrine disruption

WWE has often been reported to cause endocrine disruption in fish by estrogenic or anti-androgenic mechanisms (Rostkowski et al. 2011; Jobling et al. 2009; Routledge et al. 1998). To determine if the WWE in this study caused endocrine disruption in the roach testes, levels of selected sex steroids that have been reported previously to indicate perturbations to steroid metabolism were measured by targeted UHPLC-QTOF MS methods in fish exposed to: (i) 100 % effluent outflow (100 % WWE), (ii) 50:50 mix of effluent outflow and dilution water (50 % WWE), and (iii) dilution water alone (control). 11-KT was significantly decreased (4.27-fold, *p* = 0.01) in fish exposed to 100 % WWE compared to controls (Fig. S1a), with no significant changes to the levels of androstenedione, 11-hydroxyandrostenedione, cortisone or cortisol (Fig. S1b–e). 11-KT is the principal androgen steroid hormone in male fish (Borg 1994) and we conclude that the WWE studied here had anti-androgenic activity.

### Traditional metabolomics reveals the WWE substantially perturbs the molecular composition of the testes

**Fig. 2:**
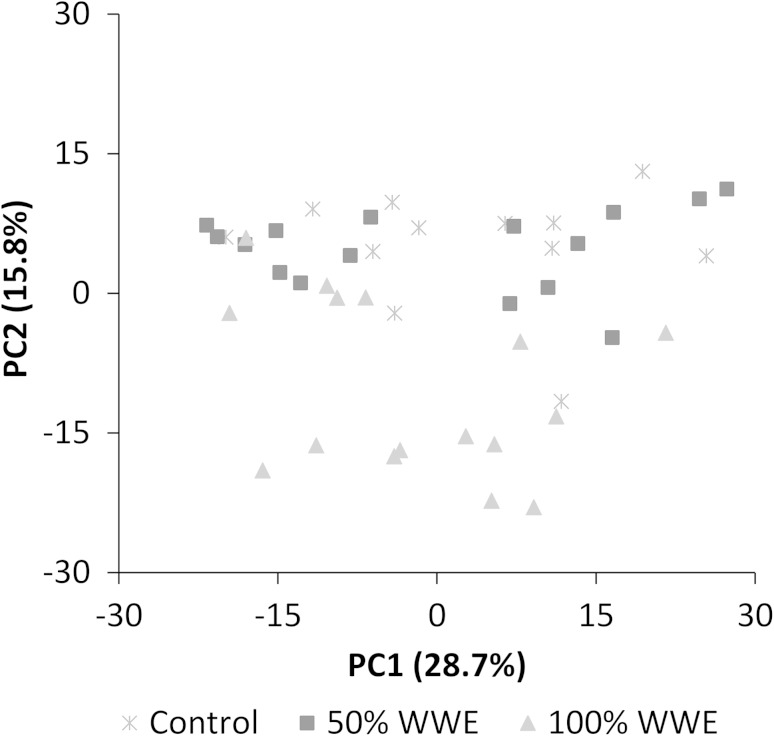
Scores plot from a PCA of DIMS measurements of the polar extracts of testes from fish that were exposed to 100 % WWE, 50 % WWE or dilution water (control) for 28 days. The control and 100 % WWE classes showed significant separation along PC2 (*t* test of the scores values: *p* = 4.4 × 10^−5^) (Color figure online)

Given the observed anti-androgenic nature of the WWE we then investigated the effects of this complex mixture on the amounts of both the polar and non-polar metabolites within the roach testes extracts, using traditional DIMS metabolomics and DIMS lipidomics analytical and computational workflows (i.e. an unbiased approach in which the peak origin, endogenous or exogenous, is unknown). The resulting PCA scores plot of the polar metabolite DIMS data demonstrated the metabolite profiles from 100 %-WWE exposed fish, but not 50 %-WWE exposed fish, were significantly different to the controls (*p* = 4.4 × 10^−5^: *t* test of the PCA scores values, Fig. 2). Of the 3,193 peaks in the entire polar DIMS dataset almost a quarter significantly changed concentrations across these three treatment groups (*q* < 0.1, Tables 1, S3). The non-polar metabolite DIMS data showed no separation of treatment groups in the PCA scores plot (Fig. S2) and only a single significantly changing peak of the 2,976 was detected (Tables 1, S4). Using these traditional analyses we would conclude that the WWE induced a considerable metabolic change in the polar metabolome of the testes, and no change to the lipidome. However, as the fish were exposed to an uncharacterised mixture of xenobiotic compounds, it is in fact not possible to infer from this dataset alone if the observed changes arise from perturbations to the endogenous biochemistry or from the accumulation of xenobiotic compounds and their metabolised products. This represents a significant and over-looked problem in environmental metabolomics.

**Table 1:** Univariate statistical analysis of the polar and non-polar DIMS datasets, comprising of all three treatment groups: control, 50 % WWE and 100 % WWE exposed roach

Dataset	Category	Total number of peaks	Number of significantly changing peaks (*q* < 0.1)
*Traditional metabolomics approach*			
Polar	All peaks	3,193	795 (24.9 %)
Lipid	All peaks	2,976	1 (0.03 %)
*Novel metabolomics workflow*			
Polar	Endogenous	3,094	734 (23.7 %)
Polar	Xenobiotics and metabolised xenobiotics	99	61 (61.6 %)
Lipid	Endogenous	2,961	0 (0 %)
Lipid	Xenobiotics and metabolised xenobiotics	15	1 (6.7 %)

The numbers of detected peaks, significantly changing peaks upon exposure, and proportion of significant peaks are shown for both a traditional metabolomics analysis and for the novel workflow reported here, the latter grouping the peaks into endogenous or xenobiotic categories. The *q* values correspond to *p* values that have been false discovery rate (FDR) corrected

### A novel workflow to distinguish endogenous and xenobiotic peaks in metabolomics datasets

To determine whether a peak originates from an endogenous or xenobiotic source, or arises from a metabolic transformation of a xenobiotic chemical, a novel computational workflow was developed that compares peak *m/z* values from DIMS analysis of the WWE against peak *m/z* values from DIMS analysis of roach testes extracts (workflow schematic shown in Fig. 1). The peak lists used in this comparison were derived from data processed using control and 100 % WWE classes only (excluding 50 % WWE) to maximise our ability to identify xenobiotic and metabolised xenobiotic peaks. Compounds within the roach testes extracts can be grouped into three categories: (1) the endogenous metabolome; (2) metabolised xenobiotic compounds, e.g. following phase two conjugation; and (3) unmodified xenobiotic compounds. Therefore, prior to comparison with the roach testes DIMS *m/z* values, the list of WWE *m/z* values were converted into a total of nine peak lists including (i) the unmodified *m/z* list; and *m/z* lists modified to take into account the following metabolic conversions and changes in electrospray ionisation ion-forms: (ii) glutathione addition, (iiii) glucuronidation, (iv) sulfation, (v) hydroxylation, (vi) methoxy addition, (vii) [M–H] to [M+OAc] conversion, (viii) [M–H] to [M+Cl] conversion, and (ix) [M–H] to [M+Na–2H] conversion. The ion-form conversions take into account the matrix differences between the sample types, and selected common metabolic conversions were used here as proof of principle. Each of these nine waste water peak lists was individually compared to the roach testes extract peak list. Peaks detected in the roach testes samples were considered to be of xenobiotic origin if they met all of the following criteria: (1) their *m/z* values were within ±1 ppm of any peaks in the WWE sample (the previously characterised mass error for our SIM-stitching approach (Southam et al. 2007)); (2) their intensity fold-change from control to 100 % WWE was ≥2-fold in the WWE sample (i.e. the xenobiotic chemical was arising primarily from the WWE and not from the dilution water); and (3) their intensity fold-change from control to 100 % WWE was ≥1.5-fold in the in roach testes sample (i.e. the xenobiotic chemical was accumulating in the testes primarily as a result of exposure to the WWE and not from the dilution water). The fold change threshold values are set deliberately low to ensure a low false negative rate; this is then followed by a second filter (accurate *m/z* comparison between datasets) that aims to reduce the number of false positive peaks.

**Fig. 3:**
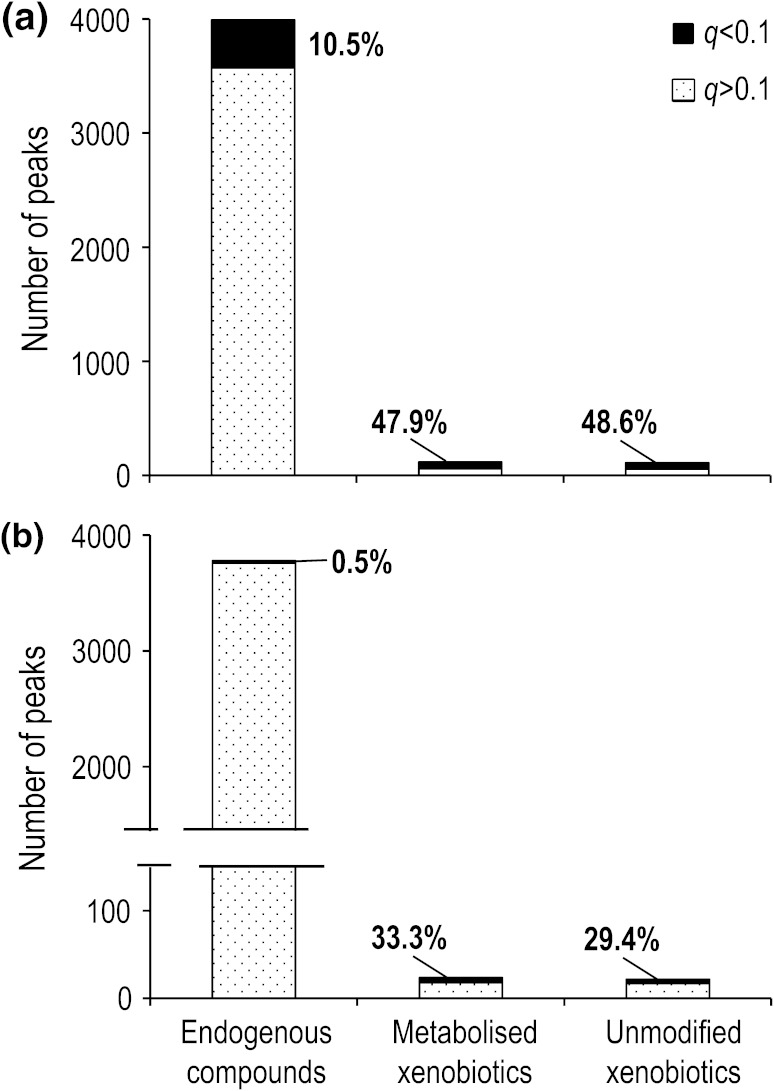
Summaries of the DIMS data comparing testes extracts from roach treated with 100 % WWE and controls only (Tables S5, S6). For each peak category, the total peak count and the number of significant peaks (*q* < 0.1) are shown for **a** polar metabolites and **b** lipids. A total of 230 peaks were flagged as being either unmodified xenobiotics or metabolised xenobiotics; of these, 114 peaks changed significantly between the control and 100 % WWE treatment groups, representing 21 % of all of the significant peaks in the “biological” DIMS dataset. This illustrates that xenobiotic-related peaks have a disproportionately high level of significance and therefore will bias and potentially invalidate the analysis and interpretation of the true biological responses unless removed

After application of these criteria to the roach testes datasets (of control and 100 % WWE only), 226 of 4,221 peaks in the polar DIMS measurements and 46 of 3,895 peaks in the non-polar measurements were determined to be either xenobiotics or metabolised xenobiotics (Fig. 3; Tables S5, S6). While these numbers of peaks may appear relatively low, they are in fact disproportionately responsible for the biochemical differences detected between the control and 100 % WWE treated fish. Specifically, the significant peaks in the xenobiotic categories are 5-fold (ca. 50 % vs 10 %, Fig. 3) and 60-fold (ca. 30 % vs 0.5 %) over-represented compared to the significant endogenous peaks, for the polar and non-polar datasets, respectively. It is not surprising that many of the peaks in the xenobiotic categories are significantly different between treatments, with the xenobiotic compounds being at higher concentrations in the effluent waste water than in the dilution control water and thus if taken up into the roach testes they would also follow this pattern. The principal findings here are that xenobiotic-related peaks can be detected in the tissue extracts, that they have a disproportionately high level of significance, and therefore they will have a major effect on the data analysis.

### Computational predictions from the DIMS datasets are validated as xenobiotics using UHPLC-QTOF MS

To validate our workflow, we then sought to identify some of the in silico predicted xenobiotic and metabolised xenobiotic compounds, i.e. to determine whether these peaks do in fact correspond to known xenobiotics and/or their metabolic products. Using UHPLC-QTOF MS, we attempted to identify several of the top 20 most intense peaks that were predicted as being xenobiotic-related in the WWE sample. From our previous investigations of similar WWE samples we expected to detect chloroxylenol, chlorophene and triclosan as they represented some of the major xenobiotics accumulating in plasma and bile of fish exposed to wastewater effluents (Al-Salhi et al. 2012; Rostkowski et al. 2011). Here, the in silico approach correctly predicted the presence of these xenobiotics and a further two surfactant (linear alkylbenzene sulfonate) metabolites. This predictive approach has demonstrated that these xenobiotics are also present in fish testes. The peaks annotated using UHPLC-QTOF MS are listed in Tables 2 and S7 and include several xenobiotics that are believed to be anti-androgenic (Rostkowski et al. 2011). This demonstrates that our computational workflow can identify successfully which peaks in the DIMS analysis of biological tissue extracts arise from known xenobiotics and their metabolic products. Furthermore, this non-targeted workflow has the potential to also discover unknown xenobiotic compounds.

**Table 2:** UHPLC-QTOF MS based identification of a selection of peaks computationally predicted as being of xenobiotic or metabolised xenobiotic origin in the direct infusion MS datasets (Tables S5, S6)

UHPLC-QTOF MS validation		Waste water effluent			Testes extract						
Name	Confirmation type	*m/z*	Peak intensity		*m/z*	Extract phase	Peak intensity		*q*	Peak modification in testes	ppm error
			Dilution water	Effluent			Dilution water	Effluent exposed			
Chloroxylenol	RT	155.02697	628	8,967	155.02696	Lipid	0	2731	1.6 × 10^−5^	None	−0.033
Chlorophene	RT & MS/MS	217.04252	0	47,570	217.04279	Lipid	0	23202	5.2 × 10^−6^	None	0.897
Chlorophene (^13^C)	RT & MS/MS	218.04591	1157	12,637	218.04614	Lipid	0	3754	2.0 × 10^−6^	None	0.704
Triclosan	RT & MS/MS	286.94392	0	49,887	366.90074	Polar	0	3917	9.1 × 10^−10^	+SO_4_	−0.011
Triclosan sulfate	MS/MS	366.90064	0	104,973						None	0.286
Triclosan (^37^Cl)	RT & MS/MS	288.94099	0	58,851	368.89780	Polar	0	4271	8.7 × 10^−9^	+SO_4_	−0.019
Triclosan sulfate (^37^Cl)	MS/MS	368.89768	0	97,743						None	0.333
Triclosan (2 × ^37^Cl)	RT & MS/MS	290.93798	0	22,895	370.89480	Polar	0	1551	2.7 × 10^−6^	+SO_4_	0.003
Triclosan sulfate (2 × ^37^Cl)	MS/MS	370.89481	0	31,439						None	−0.024
Linear alkylbenzene sulfonate (LAS) metabolite	MS/MS	357.14504	0	20,627	387.15539	Polar	68	925	2.5 × 10^−3^	+OCH_3_–H	−0.553
Linear alkylbenzene sulfonate (LAS) metabolite	MS/MS	327.13410	0	29,889						[M–H]^−^ to [M + OAc]^−^	0.413

Peaks were confirmed with standard compounds utilising UHPLC-QTOF MS retention times (RT), tandem mass spectrometry (MS/MS), or both (Table S7). The *q* values correspond to *p* values that has been FDR corrected

### Analysis of truly endogenous peaks reveals that WWE exposure perturbs energy metabolism in the testes

Knowing which peaks were predicted as xenobiotics (“novel workflow *”* section; Tables S5, S6) then allowed us to separate these peaks from the original roach DIMS datasets analysed in the “traditional metabolomics *”* section (that included all three classes: control, 50 % WWE, 100 % WWE, above; Tables S3, S4). As highlighted above, a substantially higher percentage of these xenobiotic peaks changed significantly after WWE exposure compared to the traditional data analysis that included all peaks (Table 1), highlighting the importance of identifying the origin of a peak (endogenous or xenobiotic) in order to correctly interpret the data. The endogenous peaks were now able to be interrogated independently to identify the true biological response of the roach to the WWE treatment (Table S3). While an exhaustive analysis of these changes is beyond the scope of this paper, which reports the development and validation of a novel metabolomics workflow, some of the largest and most significant metabolic changes were identified as arising from a perturbation to energy metabolism. Specifically, annotation of the significantly changing endogenous peaks in the polar dataset revealed that the [M+K–2H]^−^ ion-forms of ADP (0.68-fold, *p* = 3 × 10^−3^) and ATP (0.70-fold, *p* = 4 × 10^−4^) were significantly depleted in 100 % WWE fish compared to controls. When summing all three of the detected ion-forms for ATP and ADP ([M–H]^−^, [M+K–2H]^−^ and [M+Na–2H]^−^, Table S3) and then normalising them to levels of [AMP–H]^−^, significant perturbations to the ATP/AMP (*p* = 3 × 10^−4^) and ADP/AMP (*p* = 1 × 10^−5^) ratios were observed over the three classes (control, 50 % WWE, 100 % WWE, Fig. S3). In both cases this ratio was elevated in the 50 % WWE samples and decreased in the 100 % WWE samples demonstrating a bimodal dose response. This type of response, termed hormesis, is common in biology and we have previously observed it in the roach (Calabrese and Baldwin 2003; Southam et al. 2011). The perturbation of AMP/ADP/ATP indicated that WWE exposure disrupts the energy status of the testes tissue in the roach, and perhaps suggests an over compensation to energetic stress in the 50 % WWE group and an energy deficit following 100 % WWE treatment.

### Concluding remarks

In roach extracts analysed by the DIMS SIM-stitch method, we have demonstrated that exposure to a complex, undefined WWE led to the occurrence of xenobiotic and metabolised xenobiotic compounds in the testes. By adding a novel computational ‘module’ to the SIM-stitch workflow, we were able to group peaks into three proposed categories: (1) the endogenous metabolome, (2) metabolised xenobiotic compounds, and (3) xenobiotic compounds. We demonstrated that 3 % of the polar peaks were predicted to arise from xenobiotic origins. While relatively low in terms of fractional contribution, of these peaks more than 60 % changed significantly in WWE-exposed fish relative to controls, which most importantly accounted for 8 % of all significantly changing peaks in the exposed fish. An analysis of this dataset focusing on significantly changing peaks would result in a considerable misinterpretation without this peak origin information. This approach not only removes invalid peaks from the “biological” dataset, but also has potential to facilitate the discovery of xenobiotic and metabolised xenobiotic compounds in samples, which can then be validated by UHPLC-QTOF MS. This information on accumulation and metabolism of xenobiotics in the fish can then, in principle, be used to more fully interpret the endogenous biochemical perturbations.

## Electronic supplementary material

Below is the link to the electronic supplementary material.

Supplementary material 1 (DOCX 379 kb)
