# Supplementary material for: Distinguishing between the metabolome and xenobiotic exposome in environmental field samples analysed by direct-infusion mass spectrometry based metabolomics and lipidomics
Source: Metabolomics. 2014 Jul 15;10(6):1050–8. doi: 10.1007/s11306-014-0693-3 (PMC4213387; doi:10.1007/s11306-014-0693-3)
Supplement: Supplementary file 1 — Supplementary material 1 (DOCX 379 kb) [file 11306_2014_693_MOESM1_ESM.docx]

**Distinguishing between the metabolome and xenobiotic exposome in environmental field samples analysed by direct-infusion mass spectrometry based metabolomics – SUPPLEMENTARY INFORMATION**

Andrew D. Southam^1^, Anke Lange^2^, Raghad Al-Salhi^3^, Elizabeth M. Hill^3^, Charles R. Tyler^2^ and Mark R. Viant*^1^.

^1^ School of Biosciences, University of Birmingham, Edgbaston, Birmingham B15 2TT, UK

^2^ School of Biosciences, University of Exeter, Exeter EX4 4PS, UK

^3^ School of Life Sciences, University of Sussex, Falmer, Brighton, BN1 9QG, UK

*Corresponding author: Mark R. Viant

Phone: +44(0)121 414-2219

FAX: +44(0)121 414-5925

Email: M.Viant@bham.ac.uk

**Supplementary Figures**


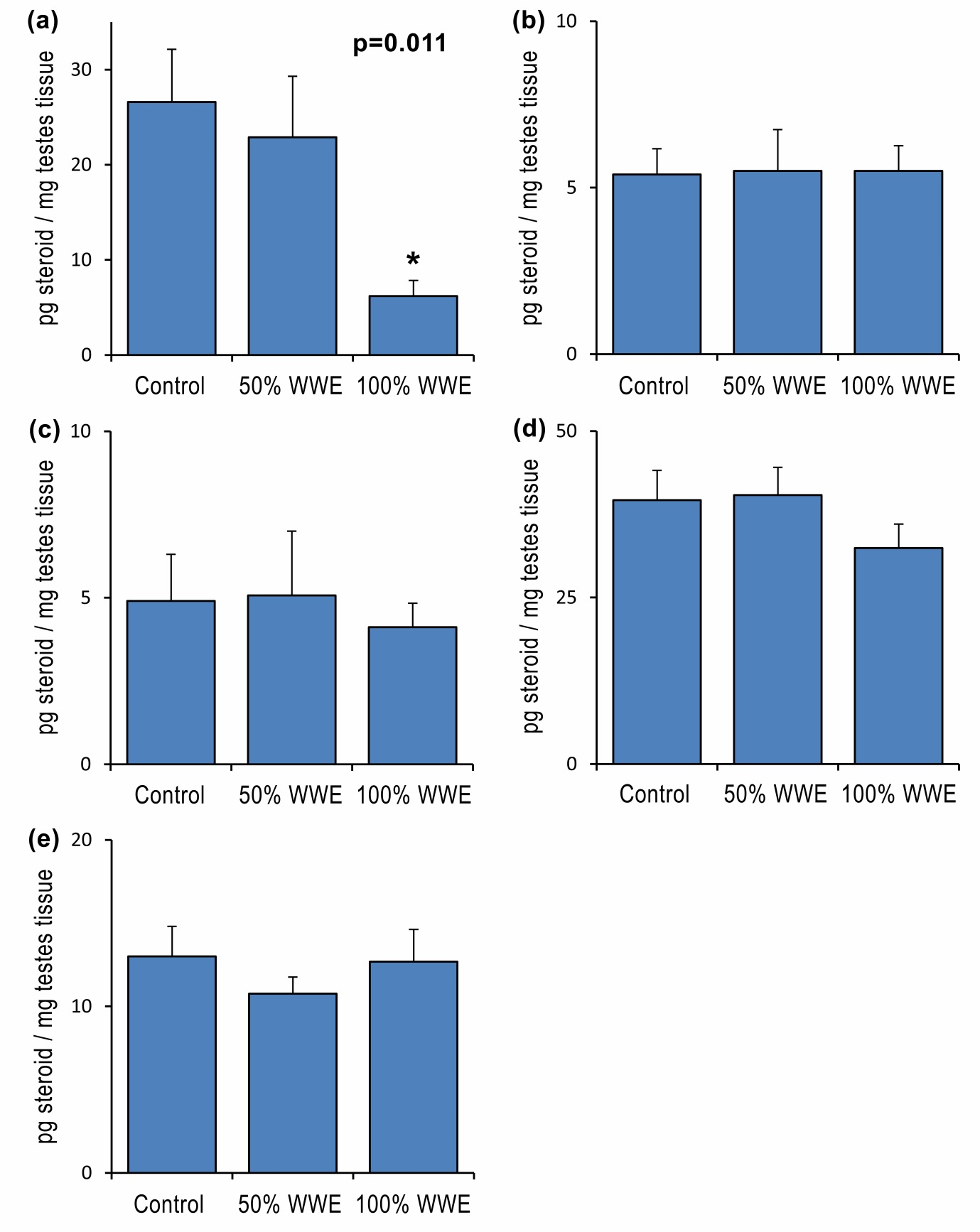


**Figure S1:** The levels of steroids found in roach testes after 28-day treatments with 100% waste water effluent (WWE) (n=16), 50% WWE (n=15) or control water (n=15). (a) 11-ketotestosterone; (b) androstenedione; (c) 11-hydroxyandrostenedione; (d) cortisol; and (e) cortisone.


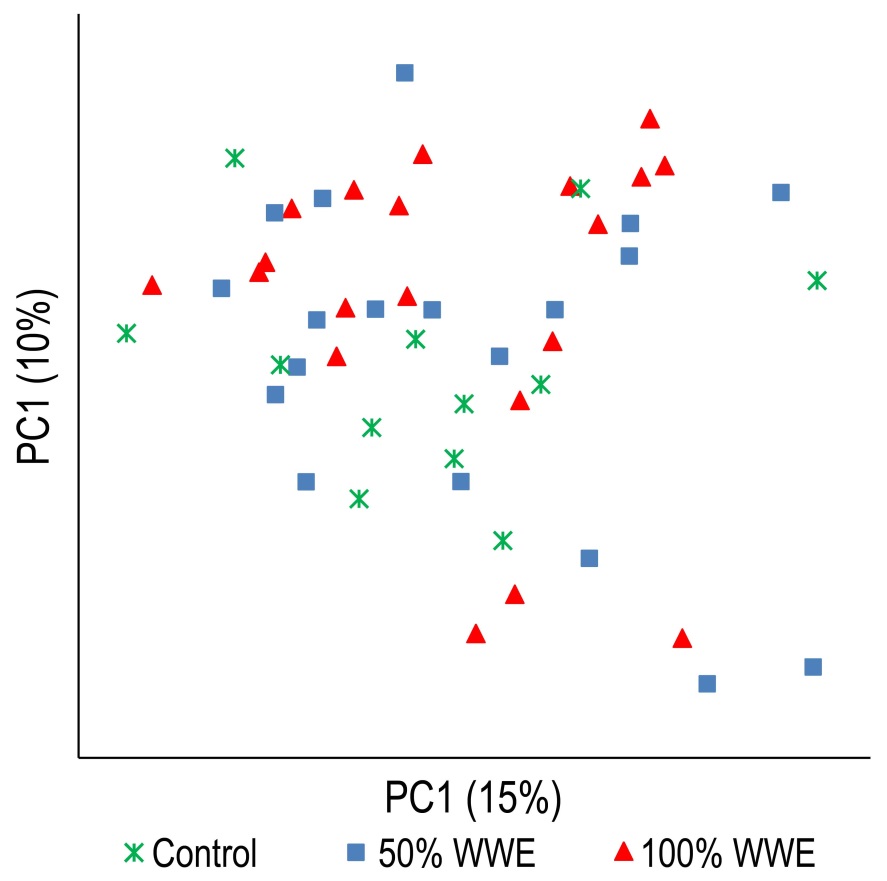


**Figure S2:** Scores plot from a principal components analysis (PCA) of direct infusion mass spectrometry (DIMS) measurements of the NON-polar extracts of testes from fish that were exposed to 100% waste water effluent (WWE), 50% WWE or upstream water (control) for 28 days.


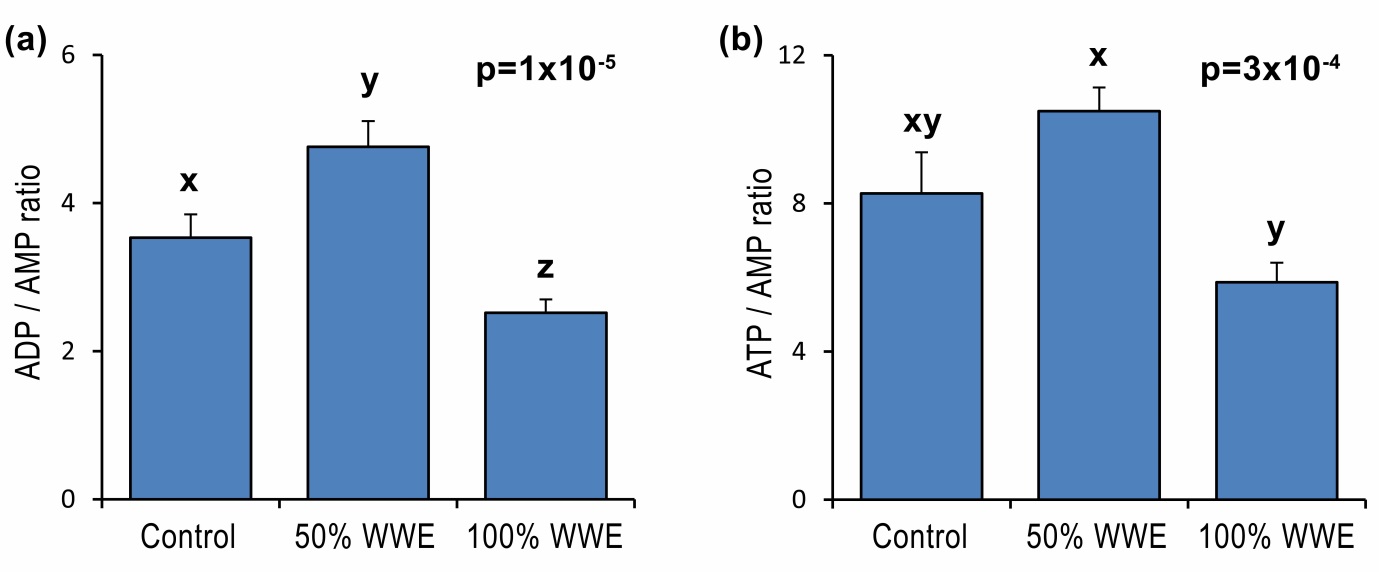


**Figure S3:** (a) ATP/AMP and (b) ADP/AMP ratios in polar extracts of roach testes tissue after 28-day treatments with 100% waste water effluent (WWE) (n=16), 50% WWE (n=16) or control water (n=12). Measurements were made by direct infusion mass spectrometry (DIMS).

**Supplementary Tables**

**Table S1:** Accurate masses of peaks used for mass spectral calibration for direct infusion negative ion lipid analysis.

| **Accurate *m/z*** | **Name** | **Ionform** | **Formula** |
| --- | --- | --- | --- |
| 480.3095651 | LPE(18:0) | [M-H]^-^ | C_23_H_47_NO_7_P^-^ |
| 582.3776447 | LPC(18:0) | [M+OAc]^-^ | C_28_H_53_NO_9_P^-^ |
| 716.5235808 | PE(34:1) | [M-H]^-^ | C_39_H_75_NO_8_P^-^ |
| 742.5392309 | PE(36:2) | [M-H]^-^ | C_41_H_77_NO_8_P^-^ |
| 744.5548810 | PE(36:1) | [M-H]^-^ | C_41_H_79_NO_8_P^-^ |
| 790.5603602 | PC(32:1) | [M+OAc]^-^ | C_42_H_81_NO_10_P^-^ |
| 816.5760103 | PC(34:1) | [M+OAc]^-^ | C_44_H_83_NO_10_P^-^ |
| 863.5655052 | PI(36:1) | [M-H]^-^ | C_45_H_84_O_13_P^-^ |
| 885.5498551 | PI(38:4) | [M-H]^-^ | C_47_H_82_O_13_P^-^ |
| 889.5811552 | PI(38:2) | [M-H]^-^ | C_47_H_86_O_13_P^-^ |

**Table S2:** DIMS analysis peaks that were common to the waste water effluent sample (WWE, not *m/z* calibrated) and testes sample (calibrated) that were also found in the extract blank. The peaks retained in this table have an *m/z* difference of ±4ppm between the two sample types. The median *m/z* difference is +1.12 ppm, with >80% of all peaks within the 0.5 – 2 ppm difference range. Uncalibrated WWE samples were then calibrated by shifting all *m/z* values by +1.12 ppm.

| **Uncalibrated WWE *m/z*** | **Calibrated testes *m/z*** | ***m/z* difference (ppm)** |
| --- | --- | --- |
| 239.07438 | 239.07349 | -3.72 |
| 483.12824 | 483.12651 | -3.58 |
| 485.14396 | 485.14224 | -3.55 |
| 399.12459 | 399.12328 | -3.28 |
| 295.18843 | 295.18757 | -2.91 |
| 112.98543 | 112.98514 | -2.57 |
| 225.11141 | 225.11085 | -2.49 |
| 313.12904 | 313.12833 | -2.27 |
| 186.11244 | 186.11203 | -2.20 |
| 171.13809 | 171.13773 | -2.10 |
| 527.19100 | 527.19009 | -1.73 |
| 388.16904 | 388.16849 | -1.42 |
| 387.16562 | 387.16512 | -1.29 |
| 446.18260 | 446.18231 | -0.65 |
| 253.14230 | 253.14216 | -0.55 |
| 137.02763 | 137.02759 | -0.29 |
| 140.98615 | 140.98611 | -0.28 |
| 537.13871 | 537.13890 | 0.35 |
| 214.04846 | 214.04855 | 0.42 |
| 201.03226 | 201.03235 | 0.45 |
| 212.16547 | 212.16559 | 0.57 |
| 119.03481 | 119.03488 | 0.59 |
| 214.14471 | 214.14484 | 0.61 |
| 212.12906 | 212.12919 | 0.61 |
| 212.07492 | 212.07505 | 0.61 |
| 210.14980 | 210.14993 | 0.62 |
| 202.10834 | 202.10847 | 0.64 |
| 168.03009 | 168.03020 | 0.65 |
| 209.15454 | 209.15468 | 0.67 |
| 206.16298 | 206.16312 | 0.68 |
| 191.05598 | 191.05611 | 0.68 |
| 175.09747 | 175.09759 | 0.69 |
| 202.98725 | 202.98739 | 0.69 |
| 201.02536 | 201.02550 | 0.70 |
| 172.14229 | 172.14241 | 0.70 |
| 186.11343 | 186.11356 | 0.70 |
| 200.12907 | 200.12921 | 0.70 |
| 171.13893 | 171.13905 | 0.70 |
| 204.13208 | 204.13223 | 0.73 |
| 270.25160 | 270.25180 | 0.74 |
| 161.04542 | 161.04554 | 0.75 |
| 173.11820 | 173.11833 | 0.75 |
| 159.10255 | 159.10267 | 0.75 |
| 172.10590 | 172.10603 | 0.76 |
| 198.14980 | 198.14995 | 0.76 |
| 171.10255 | 171.10268 | 0.76 |
| 181.15966 | 181.15980 | 0.77 |
| 194.08212 | 194.08227 | 0.77 |
| 166.05085 | 166.05098 | 0.78 |
| 255.10015 | 255.10035 | 0.78 |
| 257.23933 | 257.23954 | 0.82 |
| 158.12664 | 158.12677 | 0.82 |
| 157.12327 | 157.12340 | 0.83 |
| 157.08690 | 157.08703 | 0.83 |
| 155.10764 | 155.10777 | 0.84 |
| 187.11678 | 187.11694 | 0.86 |
| 205.15959 | 205.15977 | 0.88 |
| 155.00161 | 155.00175 | 0.90 |
| 177.04031 | 177.04047 | 0.90 |
| 259.11839 | 259.11863 | 0.93 |
| 256.23598 | 256.23623 | 0.98 |
| 255.19626 | 255.19651 | 0.98 |
| 141.01674 | 141.01688 | 0.99 |
| 259.19115 | 259.19142 | 1.04 |
| 220.14663 | 220.14686 | 1.04 |
| 217.00280 | 217.00303 | 1.06 |
| 356.28029 | 356.28067 | 1.07 |
| 215.09225 | 215.09248 | 1.07 |
| 250.14458 | 250.14485 | 1.08 |
| 145.05052 | 145.05068 | 1.10 |
| 217.14427 | 217.14451 | 1.11 |
| 280.26417 | 280.26448 | 1.11 |
| 242.17589 | 242.17616 | 1.11 |
| 284.26718 | 284.26750 | 1.13 |
| 266.18394 | 266.18424 | 1.13 |
| 221.08168 | 221.08193 | 1.13 |
| 265.18057 | 265.18087 | 1.13 |
| 228.20476 | 228.20502 | 1.14 |
| 105.01918 | 105.01930 | 1.14 |
| 87.04500 | 87.04510 | 1.15 |
| 217.10788 | 217.10813 | 1.15 |
| 247.11843 | 247.11872 | 1.17 |
| 255.23260 | 255.23290 | 1.18 |
| 101.06065 | 101.06077 | 1.19 |
| 101.02427 | 101.02439 | 1.19 |
| 151.03995 | 151.04013 | 1.19 |
| 117.05556 | 117.05570 | 1.20 |
| 283.26380 | 283.26414 | 1.20 |
| 116.07965 | 116.07979 | 1.21 |
| 115.07629 | 115.07643 | 1.22 |
| 115.03991 | 115.04005 | 1.22 |
| 114.05589 | 114.05603 | 1.23 |
| 130.05892 | 130.05908 | 1.23 |
| 96.95994 | 96.96006 | 1.24 |
| 112.98543 | 112.98557 | 1.24 |
| 120.03817 | 120.03832 | 1.25 |
| 152.03519 | 152.03538 | 1.25 |
| 133.05048 | 133.05065 | 1.28 |
| 116.07154 | 116.07169 | 1.29 |
| 231.15988 | 231.16018 | 1.30 |
| 445.18612 | 445.18670 | 1.30 |
| 130.09530 | 130.09547 | 1.31 |
| 130.05081 | 130.05098 | 1.31 |
| 129.09193 | 129.09210 | 1.32 |
| 121.03120 | 121.03136 | 1.32 |
| 135.02974 | 135.02992 | 1.33 |
| 141.01110 | 141.01129 | 1.35 |
| 122.03269 | 122.03286 | 1.39 |
| 128.03515 | 128.03533 | 1.41 |
| 135.06612 | 135.06631 | 1.41 |
| 127.00110 | 127.00128 | 1.42 |
| 340.28514 | 340.28563 | 1.44 |
| 129.05554 | 129.05573 | 1.47 |
| 142.02011 | 142.02032 | 1.48 |
| 138.01949 | 138.01970 | 1.52 |
| 144.11093 | 144.11115 | 1.53 |
| 143.07119 | 143.07141 | 1.54 |
| 143.03480 | 143.03502 | 1.54 |
| 135.04497 | 135.04518 | 1.56 |
| 139.02283 | 139.02305 | 1.58 |
| 143.10754 | 143.10777 | 1.61 |
| 341.28846 | 341.28904 | 1.70 |
| 342.26435 | 342.26495 | 1.75 |
| 338.26945 | 338.27005 | 1.77 |
| 388.26990 | 388.27059 | 1.78 |
| 328.24878 | 328.24937 | 1.80 |
| 573.21887 | 573.21993 | 1.85 |
| 536.50410 | 536.50511 | 1.88 |
| 529.46184 | 529.46284 | 1.89 |
| 555.19440 | 555.19545 | 1.89 |
| 412.34259 | 412.34340 | 1.96 |
| 473.28191 | 473.28284 | 1.96 |
| 446.18937 | 446.19028 | 2.04 |
| 564.34347 | 564.34463 | 2.06 |
| 499.18184 | 499.18294 | 2.20 |
| 530.46508 | 530.46625 | 2.21 |
| 196.07396 | 196.07441 | 2.30 |
| 125.00649 | 125.00678 | 2.32 |
| 418.21620 | 418.21719 | 2.37 |
| 432.17384 | 432.17491 | 2.48 |
| 198.08961 | 198.09024 | 3.18 |
| 388.11142 | 388.11271 | 3.32 |
| 242.08196 | 242.08284 | 3.64 |
| 323.07672 | 323.07792 | 3.71 |
| 140.01131 | 140.01184 | 3.79 |
| 212.06440 | 212.06523 | 3.91 |

**Table S3 (see attached pdf):** Data matrix showing negative ionisation direct infusion (DI) FT-ICR mass spectrometry (MS) analysis of polar testes extracts from roach exposed to control water, 50% effluent and 100% effluent for 28 days.

**Table S4 (see attached pdf):** Data matrix showing negative ionisation direct infusion (DI) FT-ICR mass spectrometry (MS) analysis of non-polar testes extracts from roach exposed to control water, 50% effluent and 100% effluent for 28 days.

**Table S5 (see attached pdf):** Data matrix showing negative ionisation direct infusion (DI) FT-ICR mass spectrometry (MS) analysis of polar testes extracts from roach exposed to control water and 100% effluent for 28 days. Data was compared with DI FT-ICR MS analyses of extracts of the water samples from control water and 100% effluent classes.

**Table S6 (see attached pdf):** Data matrix showing negative ionisation direct infusion (DI) FT-ICR mass spectrometry (MS) analysis of non-polar testes extracts from roach exposed to control water and 100% effluent for 28 days. Data was compared with DI FT-ICR MS analyses of extracts of the water samples from control water and 100% effluent classes.

**Table S7:** Metabolites identified in the 100% wastewater effluent (WWE) sample. Analysis was conducted by UHPLC-QTOFMS in negative ionisation electrospray ionisation mode.

| **FT-ICR MS (*m/z*)** | **QTOF MS (*m/z*)** | **Retention time (RT)** | **Q-TOFMS fragments** | **Identification: empirical formula of parent compound** | **Theoretical mass of identified ion** | **Identification: name** |
| --- | --- | --- | --- | --- | --- | --- |
| 155.02697 | 155.0257 | 13.19 | No fragments (low intensity peak) | C_8_H_9_OCl | 155.0264 | Chloroxylenol (RT confirmed with the STD) |
| 217.04252 | 217.0428 | 18.81 | 199.0754, 181.0657, 171.0820, 153.07 | C_13_H_11_OCl | 217.0420 | Chlorophene (RT & MSMS fragments confirmed with the STD) |
| 286.94392 | 286.9438 | 21.37 | 241.1820 | C_12_H_7_O_2_Cl_3_ | 286.9433 | Triclosan (RT & MSMS fragments confirmed with the STD) |
| 366.90063 | 366.9008 | 13.41 | 286.9429 | C_12_H_7_O_2_Cl_3_ | 366.9002 | Triclosan sulphate (identification indicated by MSMS fragment) |
| 327.13410 | 327.1277 | 7.60 | 309.20, 212.0507, 197.0271, 183.0119 170.0037 | C_16_H_24_O_5_S | 327.1266 | LAS metabolite (C10-SPC) (saturated peak) (identification indicated by MSMS fragments) |
| 357.14504 | 357.1367 | 6.10 | 339.1272, 297.1160, 183.0117 | C_17_H_26_O_6_S | 357.1372 | LAS metabolite (monohydroxylated C11-SPC) (identification indicated by MSMS fragments) |
